# Evaluating the public’s readiness to combat vector-borne disease threats in Jazan, Saudi Arabia: A cross-sectional survey

**DOI:** 10.1097/MD.0000000000039114

**Published:** 2024-07-26

**Authors:** Ahmad Y. Alqassim, Mohamed Salih Mahfouz, Abdullah A. Alharbi, Mohammed A. Muaddi, Mohammad A. Jareebi, Anwar M. Makeen, Essa A. Adawi, Mariam M. Tawhari, Atheer A. Akoor, Saud N. Alwadani, Nidaa Q. Khormi, Maram A. Sayegh, Raghad A. Mobaraki, Ghadah T. Maghfori

**Affiliations:** a Family and Community Medicine Department, Faculty of Medicine, Jazan University, Jazan, Saudi Arabia; b Surgery Department, Faculty of Medicine, Jazan University, Jazan, Saudi Arabia; c Faculty of Medicine, Jazan University, Jazan, Saudi Arabia.

**Keywords:** attitudes, knowledge, practices, prevention and control strategies, public health, Saudi Arabia, sociodemographic factors, vector-borne diseases

## Abstract

Vector-borne diseases (VBDs) pose a significant public health challenge in Saudi Arabia, particularly in the Jazan region. This study aimed to assess the knowledge, attitudes, and practices regarding VBDs among the population of Jazan and to identify factors associated with these variables. A cross-sectional survey was conducted among 642 adult residents of Jazan using a convenience random sampling technique. The survey tool consisted of 6 domains: demographics, knowledge of VBDs, preventive practices, care-seeking behavior, knowledge of specific VBDs, and attitudes towards VBDs. Data were analyzed using descriptive statistics, chi-square tests, *t* tests, ANOVA, and multivariable logistic regression. The majority of participants (60.0%) had high knowledge scores, while (75.5%) and (77.7%) had high attitude and practice scores, respectively. However, knowledge gaps were identified in specific areas, such as the transmission of leishmaniasis and Rift Valley fever. Gender was a significant predictor of both knowledge and practice scores, with males having higher knowledge and females demonstrating higher levels of preventive practices. Age, education, income, and working status were also associated with knowledge scores. The findings highlight the need for targeted interventions and educational campaigns to address the identified gaps in knowledge, attitudes, and practices. Future research should focus on exploring the effectiveness of different intervention strategies and investigating the integration of VBD prevention and control measures into existing healthcare systems. By employing a multi-disciplinary approach, evidence-based strategies can be developed to prevent and control VBDs, ultimately improving public health outcomes in Jazan and other endemic regions worldwide.

## 1. Introduction

Vector-borne diseases (VBDs) present a major worldwide health problem, resulting in significant illness and death. As per the World Health Organization (WHO), VBDs comprise more than (17%) of all infectious diseases, causing over 700,000 deaths every year.^[1]^ VBDs, impose a significantly greater burden on tropical and subtropical regions, with a particular impact on the most deprived communities.^[1]^ The Jazan region in Saudi Arabia has been recognized as a location where VBDs, such as malaria, leishmaniasis, dengue fever, and Rift Valley fever, are highly prevalent.^[2]^ Malaria control activities in the south-western regions of Saudi Arabia began in 1972, and epidemics began in the mid-1990s, raising malaria case incidence.^[3]^ Through intensive control efforts, the incidence of malaria in Jazan decreased substantially, reaching the lowest rate of 0.11 cases per 10,000 population in 2014.^[3]^ Before the 1990s, Saudi Arabia was dengue-free, but the disease spread to various regions, including Jazan, which has a high dengue incidence.^[4]^ Recent studies indicate a marked increase in dengue fever cases in Jazan, suggesting the region has become endemic for the disease, with environmental factors and insecticide resistance among *Aedes aegypti* mosquitoes potentially contributing to the high infection rates.^[2,5–7]^ However, despite the progress made in malaria control, dengue fever has emerged as a significant public health challenge in Jazan in recent years, with the region experiencing a marked increase in dengue fever incidence, suggesting the need for further epidemiological investigations and vector control measures.^[2]^ The impact of VBDs on public health in Jazan is multifaceted. These diseases not only cause significant morbidity and mortality but also place a substantial burden on the healthcare system.^[8]^ Moreover, VBDs disproportionately affect vulnerable populations, such as children, pregnant women, and low-income communities, further exacerbating health inequities.^[9]^

The climate and geography of Jazan, which includes high temperatures, humidity, and the presence of water bodies, provide ideal conditions for the reproduction of VBDs.^[2]^ In spite of the ongoing efforts made by the local health authorities to VBDs, the region is still encountering outbreaks, underscoring the necessity for efficient prevention and control strategies.^[10]^ The control and prevention of VBDs are heavily influenced by the public’s knowledge, attitude, and preventive practices.^[11,12]^ Research has indicated that insufficient understanding regarding the transmission, symptoms, and prevention of VBDs can result in inadequate implementation of preventive measures and heightened susceptibility to infection.

Several studies have examined the knowledge, attitude, and preventive practices regarding vector-borne diseases VBDs in Saudi Arabia. A study conducted in Western region of Saudi Arabia, with a sample size of 659 participants, found that the median scores for dengue fever knowledge (21 out of 35) and attitude (4 out of 5) were high. However, the median score for practice was low (3 out of 8), indicating the need for interventions to enhance public practices.^[11]^ A study conducted by Ahmed et al (2022) in the Jazan region found that out of 392 participants, the average scores for knowledge, attitude, and practices related to dengue fever were 22.77, 22.68, and 25.62, respectively. This highlights the significance of conducting thorough community-based studies to evaluate these factors and their possible predictors.^[13]^ In addition, a cross-sectional study conducted in the Jazan Province found that out of the 1070 participants, (70.5%) were aware of malaria and (67%) correctly identified fever as the main symptom. However, (59.8%) of the participants were unaware that stagnant water serves as a crucial breeding ground for mosquitoes. This highlights the necessity for focused educational initiatives.^[14]^

Although there is an increasing amount of research on VBDs in Saudi Arabia, there is a shortage of comprehensive studies that specifically examine the knowledge, attitudes, and preventive measures related to VBDs as a category of infectious diseases among the general population in the Jazan region. This region is known to have a high prevalence of diseases such as leishmaniasis and Rift Valley fever.^[11,13,14]^ Furthermore, prior research has not extensively examined the possible correlations between sociodemographic factors and the extent of knowledge, attitudes, and preventive practices in this particular context.

This study focused on Jazan because of its high prevalence of VBDs like malaria, dengue, leishmaniasis, and Rift Valley fever.^[2]^ Jazan is vulnerable to VBDs due to its unique geographical, climatic, and socioeconomic characteristics, including high temperatures, humidity, water bodies, and proximity to Yemen.^[2]^ Despite local health authorities’ efforts, the region has struggled to control VBDs, emphasizing the need to understand the population’s knowledge, attitudes, and practices (KAP).^[10]^ This study addresses critical gaps in knowledge by conducting a comprehensive cross-sectional survey among the population of Jazan, Saudi Arabia, to evaluate their understanding, beliefs, and preventive practices related to endemic VBDs. By taking a holistic approach to identifying the factors associated with these variables, the study provides valuable insights that can inform the development and implementation of targeted interventions and educational campaigns aimed at improving prevention and control strategies for VBDs in the region and beyond. The findings hold significant implications for public health in Saudi Arabia and globally, as they can guide the tailoring of interventions to address the unique needs and characteristics of the population, based on the identification of specific knowledge gaps and sociodemographic factors associated with KAP. Moreover, the study’s contributions to the growing body of research on VBDs in endemic regions worldwide can facilitate the sharing of best practices and the development of evidence-based strategies for combating these diseases. Ultimately, by enhancing the understanding of KAP and its determinants, this study supports global efforts to reduce the burden of VBDs and improve public health outcomes in vulnerable populations.

## 2. Materials and methods

### 2.1. Study design and setting

This cross-sectional study was conducted out in Jazan, a region located in the south-western part of Saudi Arabia, from December 2023 to March 2024. According to the 2022 census conducted by the Saudi General Authority of Statistics, the population of Jazan exceeds 1.5 million inhabitants.^[15]^ Jazan is 1 of the 13 provinces that make up Saudi Arabia. Jazan is known to be endemic for several VBDs such as malaria, dengue fever, leishmaniasis, and Rift Valley fever.^[2]^

### 2.2. Study population and sampling

This study was conducted among residents of Jazan, Saudi Arabia. The sample size of 661 participants was determined using Epi Info™ 7.2.4.0 software. The calculation was based on a presumed prevalence of good knowledge of 50%, a confidence level of 95%, a margin of error of (4%), and a nonresponse rate of (10%).^[16]^ A simple method of random sampling was utilized to distribute the questionnaire link, which included a unique barcode, through various social media platforms, email lists, and public places in Jazan. Participants were able to access the questionnaire either by scanning the barcode or by clicking on the provided link. Researchers were present to offer assistance to participants if necessary.

### 2.3. Data collection and study instrument

Data collectors completed a training session to familiarize themselves with the study objectives, sampling procedures, and interview techniques. This training aimed to ensure that data collection was carried out in a standardized manner. Supervision and periodic checks were carried out during the process of gathering data to guarantee the accuracy of the data and compliance with the study’s procedures. Participants were guaranteed the confidentiality and anonymity of their responses in order to promote valid answers and minimize the influence of social desirability bias.

The survey tool was created partially using a previous tool^[10]^ and underwent expert panel reviews conducted by public health professionals and infectious disease specialists to ensure both the accuracy of the content validation and face validity. The final survey comprised 6 domains: demographics (12 items); VBD knowledge (3 items); preventive practices (2 items); care-seeking behavior (2 questions); specific VBD knowledge (6 items); and attitudes toward VBDs (7 items). The questionnaire comprised a combination of multiple-choice, binary, and Likert scale questions. Prior to implementation, the questionnaire underwent a pretesting phase involving a sample of 30 participants. This was done to verify the clarity, comprehensiveness, and cultural appropriateness of the questionnaire. The instrument’s reliability, as measured by Cronbach alpha, was determined to be 0.82.

### 2.4. Statistical analysis

The characteristics of the participants were described using summary statistics, such as frequency and percentages for categorical variables. Knowledge, attitudes, and practices (KAP) scores were determined by assigning points to the correct responses and then summing the points for each domain. Participants were categorized as having either a good or poor level of KAP using predefined cutoff points. The association between demographic variables and KAP scores was examined using chi-squared tests for categorical variables. Multivariable logistic regression analysis was conducted to identify the predictors of good knowledge and practices. Corresponding multivariable odds ratios (ORs) and their respective 95% confidence intervals (CIs) were calculated. A *P* value < .05 was considered statistically significant. The data were analyzed using IBM SPSS Statistics version 26.0 (IBM Corp., Armonk, NY).

### 2.5. Ethical considerations

Ethical approval for this study was obtained from the Institutional Review Board of Jazan University (Ref No. REC-45/03/749) on September 20, 2023. Informed consent was obtained from all participants before the interviews. Confidentiality and anonymity were ensured throughout the study.

## 3. Results

The study included (642 of 661) participants (97.1%), with a slight male predominance (54.4%) (Table 1). The majority of the participants were young adults, with 43.5%, while most participants had a high educational level, with (74.1%) having completed university or higher education. More than half of the participants were single (53.0%), and (43.8%) had a monthly income of 12,000 Saudi Riyal (SR) or more. The majority of the participants lived in urban areas (61.2%), and half of them resided in coastal regions (50.6%). Regarding the history of VBDs, (11.3%) of the participants or their family members had experienced malaria, (20.8%) had experienced dengue fever, (1.3%) had experienced leishmaniasis, and (4.4%) had experienced Rift Valley fever.

**Table 1:** Sociodemographic characteristics of study population (n = 642).

Variables		N	%
Gender	Male	349	54.4%
	Female	293	45.6%
Age groups (yrs)	18 to 25 yrs	279	43.5%
	26 to 35 yrs	225	35.0%
	36 to 45 yrs	83	12.9%
	46 to 65 yrs	55	8.6%
Highest education	Intermediate and less	8	1.2%
	Secondary education	158	24.6%
	University and above	476	74.1%
Marital status	Single	340	53.0%
	Married	279	43.5%
	Divorced/widowed	23	3.6%
Monthly income (SR*)	<3000	59	9.2%
	3000 to <6000	84	13.1%
	6000 to <9000	99	15.4%
	9000 to <12,000	119	18.5%
	12,000 SR or more	281	43.8%
Mode of living	Rural	249	38.8%
	Urban	393	61.2%
Geographic location	Plain	244	38.0%
	Coastal	325	50.6%
	Mountains	73	11.4%
Working status	Working	321	50.0%
	Not-working	321	50.0%
Have you or any member of your family ever had malaria?	Yes	72	11.3%
	No	567	88.7%
Have you or any member of your family ever had Dengue fever?	Yes	133	20.8%
	No	505	79.2%
Have you or any member of your family ever had leishmaniasis?	Yes	8	1.3%
	No	628	98.7%
Have you or any member of your family ever had Rift Valley fever?	Yes	28	4.4%
	No	613	95.6%

* SR = Saudi Riyal = 3.75 US Dollar.

Table 2 presents the knowledge of VBDs among the Jazan population. The majority of participants correctly identified fever (91.3%), headache (81.2%), and muscle/joint pain (80.5%) as the main symptoms of VBDs. However, knowledge of other symptoms such as vomiting (62.8%), rash (47.2%), jaundice (42.8%), and bleeding problems (21.8%) was lower. Most participants correctly identified mosquito bites as the mode of transmission for malaria (79.0%) and dengue fever (72.3%). Regarding risk groups for severe malaria, only (5.6%) and (11.8%) of participants identified pregnant women and children under 5, respectively, as high-risk groups. Knowledge about the complications of dengue fever was also limited, with only (40.7%) of participants correctly identifying all the listed complications. Gender differences in knowledge were observed, with males having significantly higher knowledge about the transmission of malaria (*P* < .001), dengue fever (*P* < .001), leishmaniasis (*P* = .005), and Rift Valley fever (*P* = .019) compared to females.

**Table 2:** Knowledge of VBDs among Jazan population (n = 642).

Questions			All participants		Male		Female		*P* value*
			N	%	N	%	N	%	
What are the main symptoms of these VBDs?	Fever	Yes	586	(91.3)	317	(90.8)	269	(91.8)	.901
		No	5	(.8)	3	(.9)	2	(.7)	
		Don’t know	51	(7.9)	29	(8.3)	22	(7.5)	
	Headache	Yes	521	(81.2)	285	(81.7)	236	(80.5)	.935
		No	13	(2.0)	7	(2.0)	6	(2.0)	
		Don’t know	108	(16.8)	57	(16.3)	51	(17.4)	
	Muscle/joint pain	Yes	517	(80.5)	283	(81.1)	234	(79.9)	.741
		No	13	(2.0)	8	(2.3)	5	(1.7)	
		Don’t know	112	(17.4)	58	(16.6)	54	(18.4)	
	Vomiting	Yes	403	(62.8)	217	(62.2)	186	(63.5)	.037
		No	33	(5.1)	25	(7.2)	8	(2.7)	
		Don’t know	206	(32.1)	107	(30.7)	99	(33.8)	
	Rash	Yes	303	(47.2)	161	(46.1)	142	(48.5)	.467
		No	76	(11.8)	38	(10.9)	38	(13.0)	
		Don’t know	263	(41.0)	150	(43.0)	113	(38.6)	
	Bleeding problems	Yes	140	(21.8)	88	(25.2)	52	(17.7)	.061
		No	166	(25.9)	83	(23.8)	83	(28.3)	
		Don’t know	336	(52.3)	178	(51.0)	158	(53.9)	
	Jaundice (yellow skin/eyes)	Yes	275	(42.8)	142	(40.7)	133	(45.4)	.072
		No	87	(13.6)	57	(16.3)	30	(10.2)	
		Don’t know	280	(43.6)	150	(43.0)	130	(44.4)	
Malaria is transmitted by		Mosquito bites	507	(79.0)	301	(86.2)	206	(70.3)	**<.001**
		Tick bites	5	(.8)	2	(.6)	3	(1.0)	
		Sandfly bites	30	(4.7)	8	(2.3)	22	(7.5)	
		Don’t know	100	(15.6)	38	(10.9)	62	(21.2)	
Which groups are most at risk of severe malaria?		Pregnant women	36	(5.6)	19	(5.4)	17	(5.8)	.525
		Children < 5	76	(11.8)	43	(12.3)	33	(11.3)	
		Adults over 65	28	(4.4)	19	(5.4)	9	(3.1)	
		Everyone is equally affected	302	(47.0)	166	(47.6)	136	(46.4)	
		I don’t know	200	(31.2)	102	(29.2)	98	(33.4)	
Dengue fever is transmitted by		Mosquito bites	464	(72.3)	263	(75.4)	201	(68.6)	**<.001**
		Tick bites	18	(2.8)	10	(2.9)	8	(2.7)	
		Sandfly bites	32	(5.0)	5	(1.4)	27	(9.2)	
		Don’t know	128	(19.9)	71	(20.3)	57	(19.5)	
Dengue fever can cause		Bleeding problems	37	(5.8)	18	(5.2)	19	(6.5)	.119
		Severe joint pain	181	(28.2)	88	(25.2)	93	(31.7)	
		Jaundice	12	(1.9)	4	(1.1)	8	(2.7)	
		All the above	261	(40.7)	153	(43.8)	108	(36.9)	
		Don’t know	151	(23.5)	86	(24.6)	65	(22.2)	
Leishmaniasis is transmitted by		Mosquito bites	54	(8.4)	38	(10.9)	16	(5.5)	**.005**
		Tick bites	33	(5.1)	12	(3.4)	21	(7.2)	
		Sandfly bites	111	(17.3)	68	(19.5)	43	(14.7)	
		Don’t know	444	(69.2)	231	(66.2)	213	(72.7)	
Rift Valley fever is transmitted by		Mosquito bites	218	(34.0)	136	(39.0)	82	(28.0)	**.019**
		Tick bites	22	(3.4)	10	(2.9)	12	(4.1)	
		Sandfly bites	160	(24.9)	86	(24.6)	74	(25.3)	
		Don’t know	242	(37.7)	117	(33.5)	125	(42.7)	

Bold values are statistically significance *P* < 0.05.

* *P* value is based on chi-squared test.

Table 3 presents the proportion of participants who reported engaging in various preventive practices to reduce the risk of VBDs. The most common preventive actions were using insecticides/sprays (94.7%), removing standing water (93.3%), using insect repellent (92.7%), and wearing protective clothing (89.1%). The least common preventive action was clearing vegetation around the home (65.7%). Significant gender differences were observed in the use of protective clothing (*P* = .023) and keeping animals away (*P* = .021), with females being more likely to engage in these practices compared to males.

**Table 3:** Preventive practices to reduce vector-borne disease risk among Jazan population (n = 642).

Preventive actions	Proportion with (yes) reponses						*P* value*
	All participants		Male		Female		
	N	%	N	%	N	%	
Use bed nets	554	(86.3)	295	(84.5)	259	(88.4)	.156
Use insect repellent	595	(92.7)	318	(91.1)	277	(94.5)	.097
Remove standing water	599	(93.3)	322	(92.3)	277	(94.5)	.251
Use window screens	566	(88.2)	303	(86.8)	263	(89.8)	.250
Clear vegetation around home	422	(65.7)	223	(63.9)	199	(67.9)	.285
Use insecticides/sprays	608	(94.7)	330	(94.6)	278	(94.9)	.855
Avoid peak biting times	503	(78.3)	268	(76.8)	235	(80.2)	.296
Wear protective clothing	572	(89.1)	302	(86.5)	270	(92.2)	**.023**
Keep animals away	523	(81.5)	273	(78.2)	250	(85.3)	**.021**
Get vaccinated	540	(84.1)	289	(82.8)	251	(85.7)	.324

Bold values are statistically significance *P* < 0.05.

* *P* value is based on chi-squared test.

**Figure 1.**
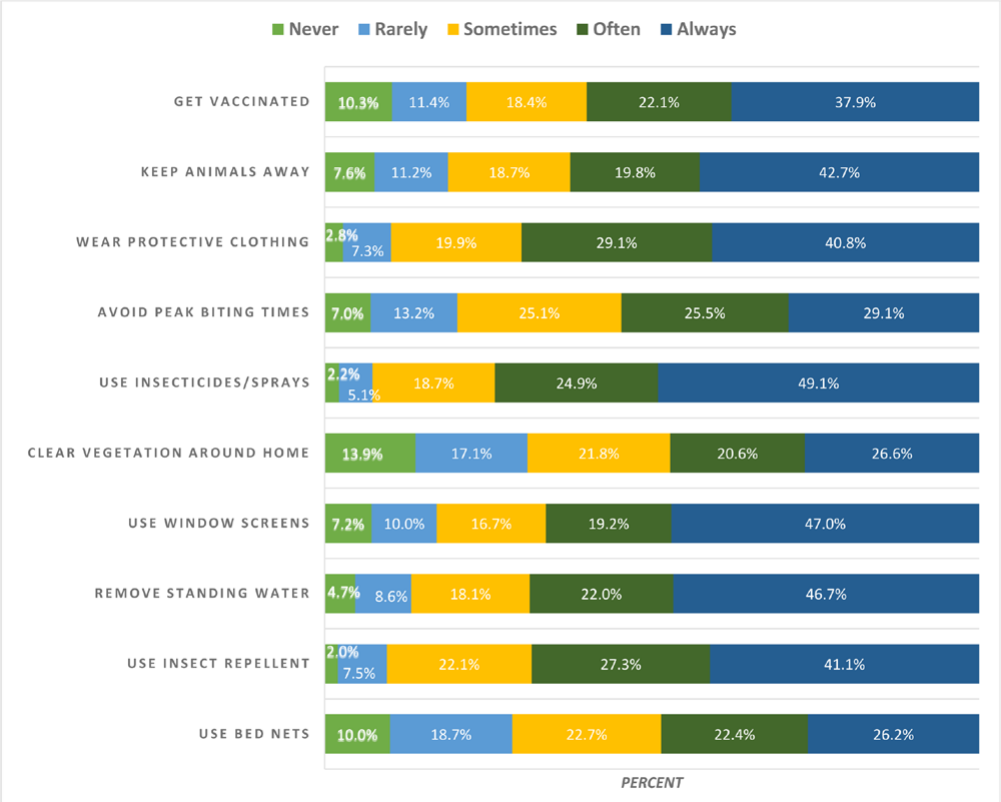
Frequency of preventive actions to reduce vector-borne disease risk among Jazan population (n = 642).

Figure 1 illustrates the frequency of preventive actions among the Jazan population. The preventive actions that were most frequently reported as “always” practiced were using insecticides/sprays (49.1%), using window screens (47.0%), and removing standing water (46.7%). In contrast, the preventive actions that were most frequently reported as “never” practiced were clearing vegetation around the home (13.9%), getting vaccinated (10.3%), and using bed nets (10.0%). The frequency of other preventive actions varied, with a considerable proportion of participants reporting “sometimes,” “often,” or “rarely” engaging in these practices.

Table 4 presents the attitudes of the study population toward VBDs. The majority of participants (72.5%) agreed that they feel at risk of getting a vector-borne disease, and (95.1%) agreed that VBDs are a serious health problem in Jazan. Most participants (85.8%) believed that VBDs can be prevented through individual actions, and (97.6%) agreed that community-level action is needed to control these diseases. A high proportion of participants (92.3%) agreed that the government is doing enough to address VBDs. Regarding symptom recognition, 87.4% of participants agreed that they would notice symptoms of a vector-borne disease quickly. However, attitudes toward traditional medicine were mixed, with (53.7%) agreeing that traditional medicine can effectively treat VBDs. No significant gender differences were observed in attitudes towards VBDs (*P* > .05 for all statements).

**Table 4:** Attitudes of study population toward VBDs (n = 642).

Statement		All participants		Male		Female		*P* value
		N	%	N	%	N	%	
I feel I am at risk of getting VBDs are a serious health problem in Jazan	Disagree^a^	126	(27.5)	66	(27.7)	60	(27.1)	.889
	Agree^b^	333	(72.5)	172	(72.3)	161	(72.9)	
VBDs are a serious health problem in Jazan	Disagree	27	(4.9)	14	(4.7)	13	(5.2)	.788
	Agree	523	(95.1)	285	(95.3)	238	(94.8)	
VBDs can be prevented through individual actions	Disagree	70	(14.2)	39	(14.6)	31	(13.7)	.778
	Agree	423	(85.8)	228	(85.4)	195	(86.3)	
Community-level action is needed to control VBDs the government is doing enough to address VBDs	Disagree	14	(2.4)	5	(1.6)	9	(3.4)	.167
	Agree	563	(97.6)	306	(98.4)	257	(96.6)	
The government is doing enough to address VBDs	Disagree	41	(7.7)	26	(9.0)	15	(6.2)	.219
	Agree	490	(92.3)	262	(91.0)	228	(93.8)	
I would notice symptoms of a vector-borne disease quickly	Disagree	54	(12.6)	30	(13.2)	24	(11.9)	.677
	Agree	375	(87.4)	197	(86.8)	178	(88.1)	
Traditional medicine can effectively treat VBDs	Disagree	203	(46.3)	114	(48.1)	89	(44.3)	.424
	Agree	235	(53.7)	123	(51.9)	112	(55.7)	

*P value is based on chi-squared test.

a Involve disagree and strongly disagree.

b Include agree and strongly agree.

Table 5 presents the association of participant characteristics with KAP regarding VBDs. Significant associations were found between knowledge scores and gender (*P* = .011), age (*P* < .001), education (*P* < .001), marital status (*P* = .016), monthly income (*P* < .001), working status (*P* < .001), and personal or family history of malaria (*P* = .013), dengue fever (*P* = .021), and Rift Valley fever (*P* = .015). Attitude scores were not significantly associated with most participant characteristics, except for a personal or family history of dengue fever (*P* = .008), with those having a history of dengue fever showing more positive attitudes. Practice scores were significantly associated with gender (*P* < .001), with females demonstrating higher levels of preventive practices compared to males. No significant associations were found between practice scores and other participant characteristics. Overall, (60.0%) of participants had high knowledge scores, 75.5% had high attitude scores, and (77.7%) had high practice scores regarding VBDs.

**Table 5:** Association of characteristics of the participants with knowledge, attitudes, and practices regarding VBDs (n = 642).

Variables		Knowledge scores			Attitude scores			Practices scores		
		Low	High	*P* value	Low	High	*P* value	Low	High	*P* value*
		N = 257	N = 385		N = 157	N = 485		N = 143	N = 499	
Gender	Male	35.5	64.5	**.011**	24.4	75.6	.949	27.8	72.2	**<.001**
	Female	45.4	54.6		24.6	75.4		15.7	84.3	
Age groups (yrs)	18 to 25 yrs	51.6	48.4	**<.001**	26.2	73.8	.711	23.7	76.3	.427
	26 to 35 yrs	33.8	66.2		24.4	75.6		23.6	76.4	
	36 to 45 yrs	32.5	67.5		21.7	78.3		15.7	84.3	
	46 to 65 yrs	18.2	81.8		20.0	80.0		20.0	80.0	
Highest education	Intermediate and less	50.0	50.0	**<.001**	50.0	50.0	.170	37.5	62.5	.522
	Secondary education	52.5	47.5		26.6	73.4		23.4	76.6	
	University and above	35.7	64.3		23.3	76.7		21.6	78.4	
Marital status	Single	45.0	55.0	**.016**	24.1	75.9	.383	23.5	76.5	.464
	Married	33.7	66.3		25.8	74.2		21.5	78.5	
	Divorced/Widowed	43.5	56.5		13.0	87.0		13.0	87.0	
Monthly income (SR)	<3000	45.8	54.2	**<.001**	27.1	72.9	.912	22.0	78.0	.683
	3000 to <6000	56.0	44.0		25.0	75.0		26.2	73.8	
	6000 to <9000	49.5	50.5		27.3	72.7		17.2	82.8	
	9000 to <12,000	32.8	67.2		23.5	76.5		22.7	77.3	
	12,000 SR or more	33.8	66.2		23.1	76.9		22.8	77.2	
Mode of living	Rural	35.3	64.7	.054	24.5	75.5	.984	24.9	75.1	.203
	Urban	43.0	57.0		24.4	75.6		20.6	79.4	
Geographic location	Plain	34.0	66.0	**.044**	21.3	78.7	.248	24.6	75.4	.505
	Coastal	43.1	56.9		25.5	74.5		21.2	78.8	
	Mountains	46.6	53.4		30.1	69.9		19.2	80.8	
Working status	Working	29.6	70.4	**<.001**	23.1	76.9	.409	21.5	78.5	.635
	Not-working	50.5	49.5		25.9	74.1		23.1	76.9	
Have you or any member of your family ever had malaria?	Yes	26.4	73.6	**.013**	18.1	81.9	.182	19.4	80.6	.547
	No	41.6	58.4		25.2	74.8		22.6	77.4	
Have you or any member of your family ever had Dengue fever?	Yes	31.6	68.4	**.021**	15.8	84.2	**.008**	18.0	82.0	.174
	No	42.6	57.4		26.9	73.1		23.6	76.4	
Have you or any member of your family ever had Leishmaniasis?	Yes	25.0	75.0	.371	12.5	87.5	.421	37.5	62.5	.293
	No	40.6	59.4		24.8	75.2		22.0	78.0	
Have you or any member of your family ever had Rift Valley fever?	Yes	17.9	82.1	**.015**	21.4	78.6	.700	14.3	85.7	.297
	No	40.9	59.1		24.6	75.4		22.7	77.3	
Overall		**40.0**	**60.0**		**24.5**	**75.5**		**22.3**	**77.7**	

Bold values are statistically significance *P* < 0.05.

* *P* value is based on chi-squared test; Figures in the Tables reflects the percentages only.

**Figure 2.**
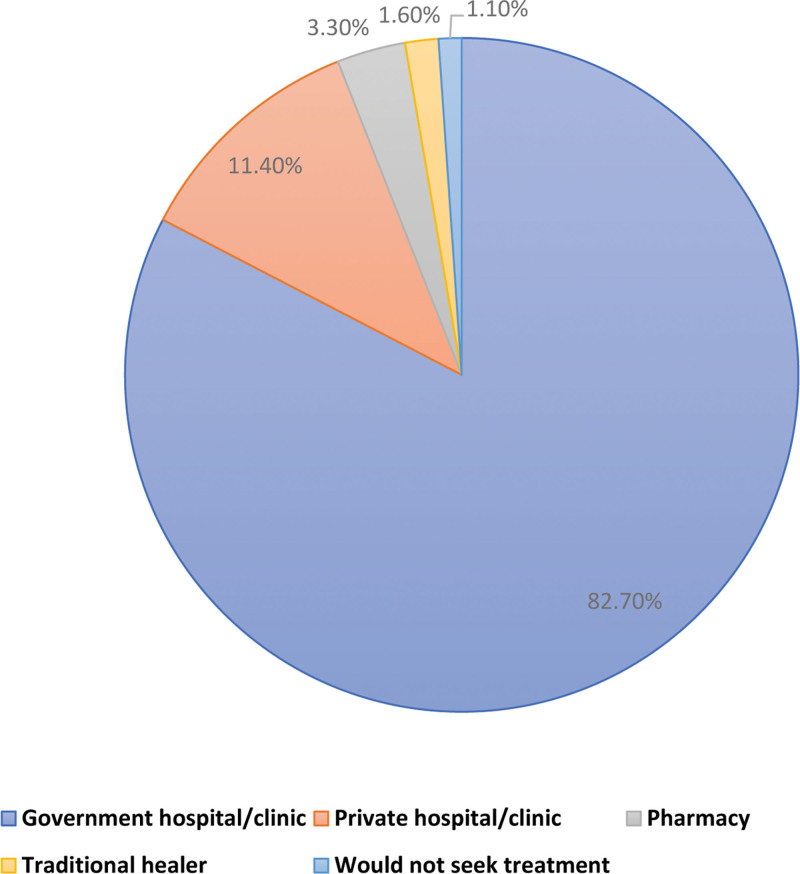
Participants care-seeking preferences in case of symptoms of a vector-borne disease (n = 642).

Figure 2 illustrates the care-seeking preferences of participants in case of symptoms suggestive of a vector-borne disease. The majority of participants (82.7%) reported that they would seek care at government hospitals and clinics, followed by private hospitals and clinics (11.4%). A small proportion of participants indicated that they would visit a pharmacy (3.3%), consult a traditional healer (1.6%), or not seek treatment at all (1.1%).

Table 6 presents the results of 2 multivariable logistic regression models examining factors associated with knowledge and practice regarding VBDs. In Model I, which assessed predictors of high knowledge scores, participants with a monthly income of 9000 SR or more (AOR = 1.9, 95% CI: 1.3–2.7, *P* < .001) and those who were working (AOR = 1.9, 95% CI: 1.3–2.7, *P* = .001) had significantly higher odds of having high knowledge scores compared to their counterparts. Gender, age, and personal or family history of VBDs were not significantly associated with knowledge scores in the adjusted model. Model II investigated predictors of high practice scores. Females had significantly higher odds of having high practice scores compared to males (AOR = 2.3, 95% CI: 1.5–3.5, *P* < .001). Participants with high knowledge scores (AOR = 1.6, 95% CI: 1.1–2.4, *P* = .020) and those with high attitude scores (AOR = 3.2, 95% CI: 2.1–4.8, *P* < .001) were more likely to have high practice scores compared to those with low scores.

**Table 6:** Multivariable logistic regression analysis of factors associated with Knowledge and practice regarding VBDs (n = 642).

Model	Variable		*COR*	95% CI		*P* value	*AOR*	95% CI		*P* value
				Lower	Upper			Lower	Upper	
Model 1 (knowledge)	Gender	Female	1				1			
		Male	1.5	1.1	2.1	**.011**	1.1	0.8	1.6	.449
	Age (yrs)	18 to 35	1				1			
		36 and above	2.1	1.4	3.2	**<.001**	1.5	0.9	2.3	.111
	Monthly income (SR)	<9000	1				1			
		9000 or more	2.1	1.5	2.8	**<.001**	1.9	1.3	2.7	**<.001**
	Work status	Not-working	1				1			
		Working	2.4	1.8	3.4	**<.001**	1.9	1.3	2.7	**.001**
	Have you or any member of your family ever had malaria?	No	1				1			
		Yes	2.0	1.1	3.4	**.014**	1.7	0.9	3.1	.083
	Have you or any member of your family ever had Dengue fever?	No	1							
		Yes	1.6	1.1	2.4	**.022**	1.5	0.9	2.3	.094
	Have you or any member of your family ever had Rift Valley fever?	No	1				1			
		Yes	3.2	1.2	8.5	**.020**	2.5	0.9	7.1	.086
Model II (practice)	Gender	Male	1				1			
		Female	2.1	1.4	3.1	**<.001**	2.3	1.5	3.5	**<.001**
	Knowledge scores	Low	1				1			
		High	1.6	1.1	2.3	**.014**	1.6	1.1	2.4	**.020**
	Attitude scores	Low	1							
		High	3.3	2.2	4.9	**<.001**	3.2	2.1	4.8	**<.001**

Model I shows predictors of high knowledge scores, while Model II illustrates predictors of high practice scores. Bold values are statistically significance *P* < 0.05.

AOR = adjusted OR, CI = confidence interval, COR = crude odds ratio, REF = references.

## 4. Discussion

This study presents important insights into the KAP concerning VBDs among the population in Jazan, Saudi Arabia. The results of our study indicate that although the majority of participants had good scores in both knowledge and attitude, with 60.0% and 75.5%, respectively, there were significant gaps in certain areas of knowledge and preventive practices. For example, there was a lack of knowledge regarding the transmission of leishmaniasis and Rift Valley fever, and the least common preventive practices were the clearing vegetation around the home. The results align with previous studies conducted in Saudi Arabia and other areas with high VBDs prevalence, which have also identified comparable gaps in knowledge and preventive practices regarding VBDs.^[11,12]^

Nevertheless, our study extends the existing literature by conducting a thorough evaluation of KAP regarding various VBDs in Jazan, a region known for its high prevalence of these diseases.^[2]^ In addition, our research reveals that the majority of participants showed a preference for public hospitals and clinics when seeking medical care. This contrasts with a previous study conducted in Ethiopia, where traditional healers were found to be the primary care providers for malaria.^[17]^ The disparity can be attributed to the greater availability and affordability of public healthcare services in Saudi Arabia in contrast to other developing countries.^[18]^

Our study revealed several sociodemographic factors that were significantly associated with KAP related to VBDs. The gender disparities in knowledge and practices related to VBDs can be attributed to the distinct societal roles and responsibilities assigned to men and women in the region. Men in traditional societies often participate more in outdoor activities and have greater access to education and information, leading to higher knowledge scores.^[19,20]^ In contrast, women frequently assume the responsibility for household duties and childcare, resulting in increased awareness and adoption of preventive measures, such as the utilization of bed nets and the elimination of standing water.^[21]^

Gender-specific roles and experiences can influence an individual’s vulnerability to VBDs and their understanding of preventive strategies, which in turn affects their knowledge and practices.^[19–22]^ Knowledge scores were significantly associated with age, education, income, and working status. Older individuals, those with higher education levels, and employed individuals had higher scores. These findings align with previous research that showed a direct correlation between socioeconomic status and health literacy.^[23,24]^ Interestingly, personal or family history of VBDs was associated with knowledge scores but not with attitude or practice scores. This suggests that while personal experience with VBDs may increase awareness and knowledge, it may not necessarily translate into improved attitudes or preventive practices.^[25]^ These findings highlight the complex interplay of sociodemographic factors in shaping KAP related to VBDs and underscore the importance of considering these factors when designing and implementing public health interventions.

The results of this study have significant implications for the implementation of public health practices in Jazan and similar environments. The identified gaps in KAP regarding VBDs emphasize the necessity for targeted interventions and educational campaigns to enhance VBDs prevention and control. Implementing community-based educational initiatives that specifically target the transmission, clinical manifestations, and prevention of VBDs, such as leishmaniasis and Rift Valley fever, could be effective in addressing the identified gaps in knowledge outlined in this study.^[26]^ It is essential to customize these programs to meet the specific requirements and preferences of the target population by considering their sociodemographic characteristics and cultural beliefs.^[27]^ Mass media campaigns, such as broadcasting radio and television advertisements, may also be used to disseminate information about VBDs to a broader audience.^[28]^ Incorporating VBDs preventive measures into primary healthcare services can guarantee that individuals receive thorough and uniform information about VBDs during regular healthcare visits.^[29]^ One possible approach is to train healthcare providers in delivering concise educational interventions and providing them with resources to support efforts in preventing and controlling VBDs.^[29]^ Our findings emphasize the need to enhance the capabilities of public hospitals and clinics in delivering excellent care for VBDs. It is crucial to make sure that these services are easily accessible and affordable for all community members.

This study has numerous strengths that enhance its validity and reliability. First, the study had a large sample size of 642 participants, determined through appropriate statistical methods, to ensure that the results accurately represent the population in Jazan.^[16]^ Second, employing a validated questionnaire that underwent pretesting and was tailored to the specific local context improves the precision and comparability of the findings.^[30]^ Third, conducting a comprehensive evaluation of KAP in relation to VBDs offers a holistic understanding of the community’s perspectives and behaviors regarding these disease.^[31]^

However, this study also has some limitations that should be acknowledged. The cross-sectional design of the study precludes causal inferences, as it only provides a snapshot of the population’s KAP at a single point in time.^[32]^ Future studies could employ longitudinal designs to investigate changes in KAP over time and establish causal relationships between sociodemographic factors and KAP.^[33]^ Additionally, the use of self-reported data may be subject to recall and social desirability bias, as participants may not accurately remember their past behaviors or may provide responses that they believe are socially acceptable.^[34]^ To address this limitation, future studies could use objective measures, such as direct observation or biological markers, to assess preventive behaviors and disease outcomes. Finally, while this study provides valuable insights into the KAP of the population in Jazan, the findings may not be generalizable to other regions or countries with different sociodemographic and cultural contexts. Future research could replicate this study in other settings to explore the transferability of the findings and identify context-specific factors that influence KAP related to VBDs.

## 5. Conclusion

This study highlights significant gaps in KAP related to VBDs among the population in Jazan, Saudi Arabia, despite overall good scores in these domains. The findings underscore the need for targeted interventions and educational campaigns to address these gaps, particularly in areas such as the transmission of leishmaniasis and Rift Valley fever and the adoption of specific preventive practices. The study also reveals important associations between sociodemographic factors and KAP, with gender, age, education, income, and working status influencing knowledge and practice scores. These insights can guide the development of context-specific strategies to prevent and control VBDs in Jazan and other endemic regions worldwide. Future research should focus on exploring the effectiveness of different intervention strategies, investigating the integration of VBD prevention and control measures into existing healthcare systems, and addressing the limitations of the current study, such as employing longitudinal designs and objective measures of preventive behaviors and disease outcomes. By adopting a multi-disciplinary approach and building upon the findings of this study, evidence-based, context-specific strategies can be developed to reduce the burden of VBDs and improve public health outcomes, particularly among vulnerable populations.

## Acknowledgments

The authors would like to thank the residents of Jazan who sacrificed their time and enthusiastically participated in the study.

## Author contributions

**Conceptualization:** Ahmad Y. Alqassim, Mohamed Salih Mahfouz, Abdullah A. Alharbi, Mohammed A. Muaddi, Mohammad A. Jareebi, Anwar M. Makeen, Essa A. Adawi, Mariam M. Tawhari, Atheer A. Akoor, Saud N. Alwadani, Nidaa Q. Khormi, Maram A. Sayegh, Raghad A. Mobaraki, Ghadah T. Maghfori.

**Data curation:** Ahmad Y. Alqassim, Mohammed A. Muaddi, Mohammad A. Jareebi, Anwar M. Makeen, Essa A. Adawi, Saud N. Alwadani, Nidaa Q. Khormi, Maram A. Sayegh, Raghad A. Mobaraki, Ghadah T. Maghfori.

**Investigation:** Ahmad Y. Alqassim, Abdullah A. Alharbi, Mohammad A. Jareebi, Anwar M. Makeen, Essa A. Adawi, Mariam M. Tawhari, Atheer A. Akoor, Maram A. Sayegh, Raghad A. Mobaraki, Ghadah T. Maghfori.

**Methodology:** Ahmad Y. Alqassim, Mohamed Salih Mahfouz, Abdullah A. Alharbi, Mohammed A. Muaddi, Anwar M. Makeen, Essa A. Adawi, Mariam M. Tawhari, Atheer A. Akoor, Saud N. Alwadani, Maram A. Sayegh, Raghad A. Mobaraki, Ghadah T. Maghfori.

**Project administration:** Ahmad Y. Alqassim, Abdullah A. Alharbi, Mohammed A. Muaddi.

**Resources:** Ahmad Y. Alqassim, Mohammed A. Muaddi, Mohammad A. Jareebi, Anwar M. Makeen, Mariam M. Tawhari.

**Software:** Ahmad Y. Alqassim, Abdullah A. Alharbi, Mariam M. Tawhari, Nidaa Q. Khormi.

**Supervision:** Ahmad Y. Alqassim, Mohamed Salih Mahfouz, Abdullah A. Alharbi, Mohammed A. Muaddi, Anwar M. Makeen, Essa A. Adawi

**Validation:** Ahmad Y. Alqassim, Mohammed A. Muaddi, Mohammad A. Jareebi, Anwar M. Makeen, Nidaa Q. Khormi.

**Writing – original draft:** Ahmad Y. Alqassim, Mohamed Salih Mahfouz, Abdullah A. Alharbi, Mohammed A. Muaddi, Mohammad A. Jareebi, Anwar M. Makeen, Essa A. Adawi, Atheer A. Akoor, Saud N. Alwadani, Nidaa Q. Khormi, Maram A. Sayegh, Raghad A. Mobaraki, Ghadah T. Maghfori.

**Writing – review & editing:** Ahmad Y. Alqassim, Mohamed Salih Mahfouz, Abdullah A. Alharbi, Mohammed A. Muaddi, Mohammad A. Jareebi, Anwar M. Makeen, Essa A. Adawi, Mariam M. Tawhari, Atheer A. Akoor, Saud N. Alwadani, Nidaa Q. Khormi, Maram A. Sayegh, Raghad A. Mobaraki, Ghadah T. Maghfori.

**Formal analysis:** Mohamed Salih Mahfouz, Mohammad A. Jareebi.

**Visualization:** Mohammad A. Jareebi, Atheer A. Akoor, Saud N. Alwadani.
